# In Defence of Mothers

**Published:** 1920-04-24

**Authors:** 


### IN DEFENCE OF MOTHERS.

By A SOCIAL WORKER.

The present writer does not know who is responsible for the recent organisation of a society calling itself the Mothers' Defence Association, and established, apparently, in order to protest against the work of the health visitor and other child-saving agencies. The promoters have certainly taken a serious step. It is only too easy to arouse in the minds of working women so strong a feeling against "interference" with conditions which are responsible for our heavy death-rate and damage-rate that the action of the Ministry of Health and of the Mother School and Infant Welfare Centre would be practically stultified. Then, indeed, those who had attained such an end would have cause for self-congratulation on a victory for "Liberty," which not very remotely suggests the paradise of Bolshevism.

### The Saving of Infant Lives.

After all, the saving of infant lives is a modern blessing. A writer lately remarked on the "procession of cradles and coffins" common in well-to-do homes in the days when John Colet was the only one to survive of a family of twenty-three. Henri IV. of France owed his life to the grandfather who rescued him from mismanagement in a royal nursery and sent him to be reared by a Pyrenean farmer's wife. Miss Alice Clark has shown in a study of English industrial life that very few children of the working classes lived to grow up in the eighteenth century; while as to the needlessly blinded, the irreparably injured by measles, the pneumonia cases resultant from mothers carrying infants in arms to the public-house at night, the overlaid babes of drunken women—these are but a few of the categories of methods by which the massacre of the innocents has been proceeding in the last few generations and in our own.

### The Working-class Mother.

But certainly there was never a period in which the working-class mother deserved so much sympathy. Her life—the term "working class" covers, of course, women living in very varied degrees of comfort—is made harder by the raised standards of decency and good appearance. Once the "gutter child" played, ragged and filthy, and learnt, or did not learn, to read and to write a little before going into some kind of labour. Now the child must be fit to take its place in school with some degree of decent grooming and dressing. That invisible tyrant, who is Public Opinion, sets a certain standard for her street of appearances in the home itself; however closely she may curtain her windows, she, and everyone else, knows of its existence. A higher idea of the care needed by little children has somehow filtered through to her and burdens her with a sense of responsibility from which her mother did not suffer. It is, in fact, the contagion of the awakened conscience of the community. For how much of the deadness of that conscience in the past has not a wrong type of religion been responsible? "It was God's will," said the mourners who left a child's grave; "we must be resigned, it is now a little angel in heaven." A more robust Christianity is now sharply traversing such feeble religiosity by quoting the Master's own saying, that it is not the will of the Father that one of these little ones should perish. And we may surely, from the most religious point of view, declare that "our God is marching on" when we see that the powerful combination movement of our day is affecting the people of all others who appeared "most unclubbable." Mothers' Unions, Village Councils, Women Citizens' Associations, Women's Co-operative Societies, Women's Institutes are everywhere binding the once isolated mothers into bodies which are gathering momentum every day, and will presently exercise an influence of which the vote is only one symptom. In bringing pressure to bear upon local councils for sanitary and other social improvements these bodies have already done much good work.

It is the more necessary that the public opinion which they inform and represent shall be an enlightened one, and that their members shall be in touch with that official work which exists to educate and keep them. How otherwise shall our social problems ever be solved? The causes of death-rate and damage-rate of infants lie in the home, and can only there be abolished. If the Housing Question is to be answered you must not only abolish the slum, but abolish the slum-maker. There are people who would convert Buckingham Palace or the Taj Mahal into slum dwellings by their modes of living. If the Drink Question is to be answered by the raising up of a sober nation that can enjoy its glass in the spirit of Anacreon or of the Old Testament vintage-gatherer, it must be by well-nourished families in homes where domestic economy is a happy reality. While as to the dreary subject of the falling birth-rate and the unwelcomed and unfathered child, that problem can only cease to trouble when the children are welcomed to homes where forethought and hope and pride have made their place assured. That is the business of women, no doubt, and must continue to be so, no matter how many new careers are opened to them. But they cannot do it properly without the help of their men folk.

### The Minutiæ of Home-rearing.

But the over-burdened mother sees only dimly, if at all, that the redemption of the nation lies in her hands. It is a great thing, and it is the very purpose of the health visitor's and the Care Committee worker's existence to uplift her trivial round and common task to glorious "worth-whileness" by showing her that each minute act is a stone rearing the fabric of a noble building. Modern discoveries in medicine point out every day some way in which the minutiæ of home-rearing are seen to involve great issues. For instance, it has lately been pointed out in The Hospital that the unclean head of a child may lead to the tuberculous neck gland, and thence to the full force of the white scourge. Right food, sufficient sleep, attention to physical habits, simple cleanliness, the domestic discipline of obedience, helpfulness, truth-telling, honesty, and those first ideas of religion which permanently colour the spirit—these are vital things and are not given to the young citizen by Acts of Parliament or Government officials. They are gained or lost according to the mother's knowledge and good-will. Her worst enemy—and the children's—is her ignorance or indifference.

To inform her mind, to stiffen her will, and to link her home work with outside agencies that can help her and befriend the children is the task of the official visitor. It is one that requires great tact and sympathy, as well as knowledge. It is essential to our national reconstruction and progress. If there is kindly intention on the part of persons trying to stultify this work, the mother has good cause to cry, "Save me from my friends!"

